# Positron kinetics in an idealized PET environment

**DOI:** 10.1038/srep12674

**Published:** 2015-08-06

**Authors:** R. E. Robson, M. J. Brunger, S. J. Buckman, G. Garcia, Z. Lj. Petrović, R. D. White

**Affiliations:** 1 College of Science, Technology and Engineering, James Cook University, Townsville QLD 4810, Australia; 2 School of Chemical and Physical Sciences, Flinders University, Adelaide, SA 5001, Australia; 3 Institute of Mathematical Sciences, University of Malaya, 5063 Kuala Lumpur, Malaysia; 4 Research School of Physics and Engineering, Australian National University, Canberra, ACT 0200, Australia; 5 Instituto de Física Fundamental, CSIC, Madrid E-28006, Spain; 6 Institute of Physics, University of Belgrade, Zemun, Belgrade 11080, Serbia

## Abstract

The kinetic theory of non-relativistic positrons in an idealized positron emission tomography PET environment is developed by solving the Boltzmann equation, allowing for coherent and incoherent elastic, inelastic, ionizing and annihilating collisions through positronium formation. An analytic expression is obtained for the positronium formation rate, as a function of distance from a spherical source, in terms of the solutions of the general kinetic eigenvalue problem. Numerical estimates of the positron range - a fundamental limitation on the accuracy of PET, are given for positrons in a model of liquid water, a surrogate for human tissue. Comparisons are made with the ‘gas-phase’ assumption used in current models in which coherent scattering is suppressed. Our results show that this assumption leads to an error of the order of a factor of approximately 2, emphasizing the need to accurately account for the structure of the medium in PET simulations.

Positron emission tomography (PET) is an established technology for pinpointing abnormalities in living tissue^1^,^2^. However, its accuracy is fundamentally limited by the fact that high energy (*ε*  100 keV) positrons emitted by the source (in the region of the abnormality, such as a tumour) must slow down over a finite distance, or “range”, to sufficiently low energies (1 eV  *ε*  100 eV in many cases) to allow formation of positronium (*Ps*), and the subsequent, virtually immediate emission of signature back-to-back gamma rays, which form the image in the external apparatus^2^. Each point in this imaging apparatus is therefore displaced from the position in the source at which the positron actually originates and, since there is a distribution of such displacements due to the randomizing effect of the various scattering processes, the overall image is somewhat blurred. This fundamental limitation on spatial resolution has long been recognized^1^,^2^,^3^. The physical limit of spatial resolution in PET is ultimately determined by three factors - positron range, annihilation photon non-collinearity and intrinsic detector resolution^4^, and as detailed by Levin and coworkers^5^ “of these other factors (including non-collinearity) that contribute to resolution broadening, perhaps the most uncertain, poorly understood, and, for certain isotopes, the most dominant effect is from positron range”. Foremost among our concerns, and the main focus of this study, is the synergy between key *low energy* (<100 eV) scattering processes using the best available set of positron-water cross-sections, and simultaneous coherent, multiple scattering of positrons due to the structured nature of the soft condensed matter medium. Although estimates of positron range using Monte Carlo simulations can be found in the literature^3^, these papers have so far effectively treated the condensed medium as a structureless gas of the same density, i.e., the synergetic effects at low energies are suppressed and not surprisingly the estimates have a significant error. On the other hand a kinetic theory of positrons in high density soft-condensed matter has recently been developed^6^,^7^ and this has paved the way for a better understanding of the positron physics at play at low energies in a PET environment. The present study represents the first step in such an investigation. We should highlight that the formalism developed here is also readily adaptable to other important applications where the transport of charged particles in tissue is important e.g. electron induced damage from ionizing radiation^8^, ion beam therapy^9^, and deep space cosmic rays effects on astronaut safety^10^.

While we readily acknowledge that the positrons emitted from the radioactive tracer source may be relativistic near that source, they are nevertheless rapidly slowed or thermalized by collisions in the bulk of the medium and so it is reasonable to use a non-relativistic form of the Boltzmann equation to describe their behaviour,


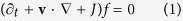


in the first instance. The collision operator *J* of Ref. 6 accounts rigorously for the structure of the medium and associated coherent scattering, and includes the various types of interaction of positrons with the constituent molecules. This equation is to be solved for the positron phase space distribution function *f*(r, v, *t*), and all quantities of physical interest then follow, after appropriate integration over velocities v, as functions of distance from the source, e.g., the rate of loss *R*_*Ps*_ of positrons by Ps formation. The positron range may then be defined in terms of some characteristic property of the profile, such as the point where *R*_*Ps*_ reaches a maximum.

Mutual interaction between positrons is negligible at the low densities involved, and hence Eqn. (1) is linear in *f*: it is effectively a “swarm” of positrons, and so the same analytical and numerical techniques which have been developed for low density, low energy electron swarms in gases may be readily adapted to the present problem. There are of course, important differences in detail: condensed matter has structure and positrons are scattered coherently from many molecules, and in a PET environment, positrons have a much wider range of energies than one encounters in electron swarm experiments. These qualifications aside, it is clear that electron kinetic theory may be used as a platform for the development of positron kinetics in PET, and this article exploits established results wherever possible.

Before proceeding with this task, it is emphasized that the current analysis is concerned with the behaviour of low energy positrons up to the point of Ps-formation, and is not meant to be viewed as a complete analysis of the PET process.

## Kinetic Theory Treatment of an Idealised PET Environment

### The model

**Figure 1:**
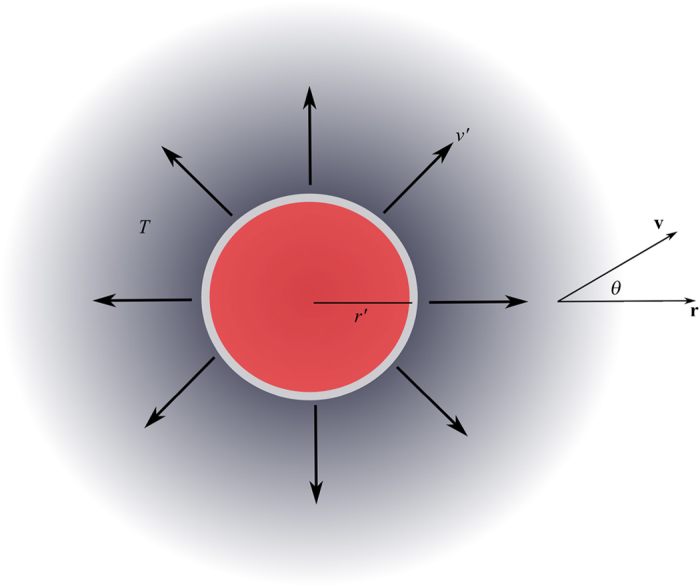
Schematic for the idealised PET model. A spherical source of radius *r*′ emits positrons isotropically with a range of speeds *v*′ at a steady state into a medium of temperature *T*.

To simplify the analysis and elucidate the essential physics, we take an idealized, spherically symmetric situation in which high energy positrons of mass *m* and charge *e* are emitted isotropically at a steady rate from a spherical source into an infinite, spatially homogeneous soft matter medium at temperature *T* (see Fig. 1). The model also assumes that a steady state has been attained whereby there is a balance between the rate at which positrons are produced at the source and the rate at which they are lost (by direct annihilation and *Ps* formation) in the medium.

Positrons are quickly slowed to lower energies by elastic, inelastic and ionizing collisions with the constituent molecules of mass *M*. The corresponding cross sections, *σ*_*m*_, *σ*_*inel*_, *σ*_*a*_ and *σ*_*Ps*_ respectively are all incorporated in the collision operator *J* (see ref. 6), where the effects of the structure medium are encapsulated in the static structure factor *S*(*Q*) (*Q* is the momentum exchange), which is the Fourier transform of the pair correlation function, *g*(*r*) (*r* is the spatial distance from the molecule). The important elements are detailed below.

### Solution of Boltzmann’s equation - The generalised eigenvalue problem

Since 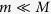, elastic collisions effectively randomize positron velocities, and *f*(r, v, *t*) quickly becomes nearly isotropic in v-space. Thus to a first approximation the spherical harmonics representation may be truncated to two terms,


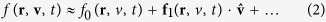


as is common in electron kinetic theory^11^,^12^. It is emphasized, however, that a “multi-term” analysis will be necessary when high precision is required. This expansion is substituted into Eqn. (1) and, the scalar component, *f*_0_, is found to be given by


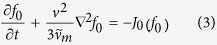


while the vector component **f**_1_ is 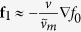. The scalar part of the collision operator is


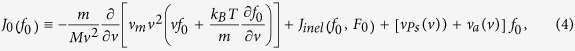


in which the first term on the right hand side arises from elastic collisions, while the other terms account for inelastic collisions, *Ps* formation and annihilation respectively and *k*_*B*_ is Boltzmann’s constant. The collision frequencies *ν*_*m*_, *ν*_*Ps*_ and *ν*_*a*_ for momentum transfer, *Ps* formation and direct annihilation, respectively, are related to the corresponding cross sections by *ν*(*v*) = *Nvσ*, where *N* is the number density of molecules in the medium. The structure-modified collision frequency 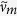 is defined in equation (6) below. Collision rates for inelastic processes (including direct ionization by positron impact) also appear in the inelastic collision operator, *J*_*inel*_, which is of the same form as for electrons^13^:


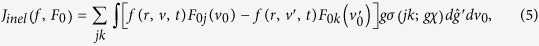


where *σ*(*jk*; *gχ*) is the differential cross section for the scattering process 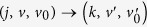 and *j*, *k* represent internal states of water molecules, with 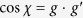 where *g* represents the relative velocity in the collision. The distribution of neutral molecules 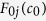 is a Maxwell-Boltzmann distribution for neutrals with internal state *j*. Due to the high density of liquid water, the de Broglie wavelength of the positron will eventually become of the order of the average inter-particle spacing ~*N*^−1/3^, and in this regime, the charged particle is best viewed as a wave that is coherently scattered from the various scattering centres in the liquid. At higher energies, such effects are minimized and the binary scattering approximation suffices. Importantly, the structure of the medium enters solely through the ‘structure-modified’ momentum transfer collision frequency, 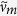, and cross-section 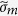
6:


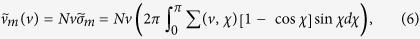


where


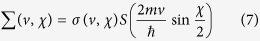


represents an effective structure modified differential cross-section of the elastic differential cross-section *σ*(*v*, *χ*) accounting explicitly for coherent scattering effects. Here *S* is the static structure factor as discussed above.

The statement of the mathematical problem is completed by specifying boundary conditions on *f*_0_ and *f*_1_ at the surface of the source in Eqn. (3), as discussed below.

Equation (3) is separable in variables, and thus by writing 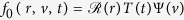 we obtain


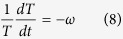



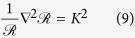



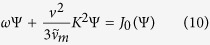


in which *ω* and *K* are separation constants. Eqn (10) is actually a special case of the general eigenvalue problem^14^,^15^ in *v*-space yielding a discrete family of ‘dispersion’ relations,


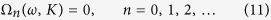


One fixes *either ω* or *K*, consistent with the nature of the problem, using boundary conditions and/or physical constraints, and the other quantity then follows. In the present article, we assume a steady state situation by specifying *ω* = 0. The allowed wave numbers *K*_*n*_ could in principle be found from Ω_*n*_(0, *K*) = 0, but in most cases it is more practical to find eigenvalues and eigenfunctions Ψ_*n*_(*v*) by solving Eqn (10) directly with *ω* = 0.

For spherical symmetry, 
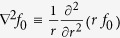
 and Eqn. (3) admits solutions of the form 
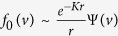
, where *K* is a constant and Ψ(*v*) is a function of speed. These quantities are found as the eigenvalues and eigenfunctions respectively of the problem


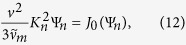


where *n* = 0, 1, 2, … is an integer enumerating the eigenvalues, which are generally found to form a discrete, real set. The most general solution of Eqn. (3) is then a linear combination of all possible modes,


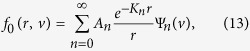


where *A*_*n*_ are constants to be found from the boundary conditions. The vector part of the distribution function is then,


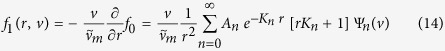


directed in the radial direction.

Similar eigenvalue problems occur naturally in the kinetic theory of gaseous electron swarms^14^,^15^. For soft condensed matter, the only difference is that the structure-modified collision frequency 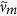 replaces the momentum transfer collisions frequency *ν*_*m*_ in the left hand side of (14). The solution of the dual eigenvalue equation,


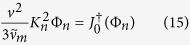


for the corresponding dual eigenfunctions Φ_*n*_ is required to effect the complete solution. The adjoint 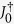 of the collision operator in Eqn. (4), is defined such that


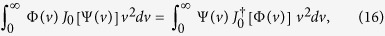


for any functions Ψ(*v*) and Φ(*v*) of positron speed *v*. The adjoint of the elastic component of the collision operator, is given by


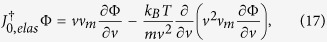


but explicit expressions for the other collision terms are not required.

Numerical solution is generally required. Only for the special model of a swarm in a gas, undergoing elastic collisions only, with constant 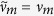, can an exact, analytic solution be found. By adapting the work of Parker^16^ we find that (apart from a constant) the eigenvalues are given by


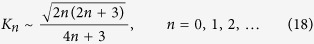


and the eigenfunctions may be written in terms of Laguerre polynomials. This result is useful in two respects:

1. As a benchmark for testing the accuracy of numerical solutions of (12) for more realistic cases; and,
2. It illustrates an important and seemingly general property of the wave number spectrum, namely that it becomes *dense* at larger values of *n*, *K*_1_ ~ 0.451, *K*_2_ ~ 0.481, *K*_3_ ~ 0.489, …, *K*_∞_ ~ 0.5.

Some general mathematical details are now given below in order to facilitate the eventual calculation of positron range in terms of the eigenvalues.

### Properties of eigenfunctions and an identity

Eqns (12) and (15) together yield the following orthogonality relation:


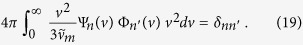


If the eigenfunctions are assumed to form a complete set in speed space, then the following closure relation follows


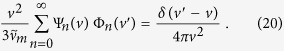


As detailed in the Appendix, 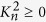 and thus the eigenvalue spectrum *K*_*n*_ consists of pairs of *real* numbers of the same magnitude, but of opposite sign. In order that the solution (13) remains physical as *r* → ∞, only the non-negative part of the spectrum *K*_*n*_ ≥ 0 contributes. Since elastic and inelastic collisions conserve positron number, integration of the first two terms of Eqn. (4) over all speeds yields zero identically. Thus integration of Eqn. (12) over all speeds yields


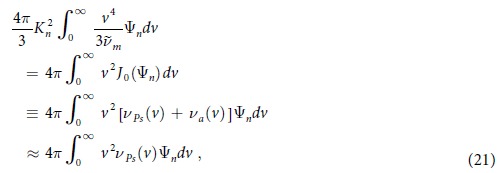


the last approximation following from the fact that annihilation is generally dominated by *Ps* formation. This identity proves useful for evaluating the *Ps* formation rate.

To make a connection between the general properties and the problem at hand, we must consider the geometry and boundary conditions of the situation.

### Boundary conditions

The constants *A*_*n*_ are found from boundary conditions at the source as follows. Let 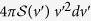 be the number of positrons emitted by the source per unit time, from its entire surface of area 4*πr*′^2^, with speeds in the range *v*′ to *v*′+*dv*′, into the surrounding medium. In the present isotropic model, positron velocities *v*′ at the source are assumed to be everywhere directed radially outward from the source at *r* = *r*′. The *exact* boundary condition is a statement that the radial flux of positrons away from the surface is equal to the number of positrons produced by unit area of the surface per unit time, at all speeds *v*′, i.e.,


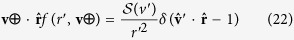


for all velocities directed outwards, i.e, 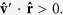 However, it is impossible to apply such an exact condition to any full-range truncated spherical harmonic representation of the distribution function, whether via the two-term formula (2), or indeed higher order “multi-term” expansions^17^,^18^. Some approximation is therefore always necessary and to that end, and in the interests of simplicity, we follow a procedure established by Mark^18^, and simply integrate Eqn. (22) over all directions to obtain


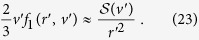


Eqn. (14) is substituted into the left hand side of this equation, which is then multiplied by 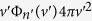, and integrated over all *v*′. The orthogonality relation (19) is then applied to yield


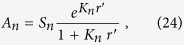


where


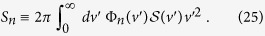


Upon substitution into Eqn. (13) we then find


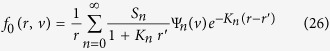


from which follows the *Ps* formation rate per unit volume:


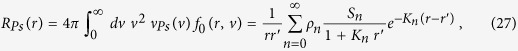


where


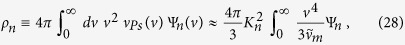


where the last step follows from integrating (12) over all speeds, noting that the particle conserving terms vanish, and identifying that 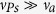.

### Positron range

The direct contribution to the positron range arises from the motion of the emitted positron through to its formation of positronium. There is an additional indirect contribution to the positron range that arises from the subsequent motion of the Ps, often involving the dissociation of Ps into a positron and electron if the Ps has a kinetic energy greater than its binding energy. This is not considered in this study. Further, the actual process of Ps-formation in liquids has been proposed through the recombination of the positron with ejected electrons in the terminus of the positron tract - the so-called blob model^19^,^20^. Here we consider only positrons involved in the direct Ps-formation, arising from collisional processes, at a rate described by (27). This quantity has a maximum when


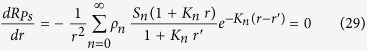


and the value of *r* obtained by solving this equation is an estimate of the positron range.

For practical purposes, one has to approximate the infinite sums in Eqns (27) and (29) by truncating them to finite size *n*_max_. In a rigorous investigation of the complete spatial profile *R*_*Ps*_(*r*), *n*_max_ would be incremented, and the whole process repeated until some convergence criterion is satisfied. However, a less stringent approach suffices for present purposes, where the aim is limited to finding the position of the maximum in *R*_*Ps*_, using Eqn. (29). Note also that the contribution from the *n* = 0 mode is negligible, since *K*_0_ is very small (see Table 1), and hence by Eqn. (28) *ρ*_0_ ≈ 0. Thus in what follows, we retain only the *n* = 1,2 modes in the summation, and write


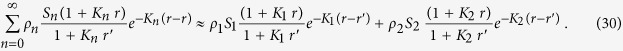


The respective contributions to these terms can be found from solutions {*K*_*n*_, Ψ_*n*_} of the eigenvalue problem (12).

Although a typical source may be small with *r*′ ≈ 10^−2^ *m*, the eigenvalues *K*_*n*_ are nevertheless sufficiently large for *n* > 0 (see Table 1) so that 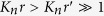, and consequently Eqn. (30) simplifies to:


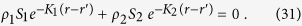


This is the equation we work with below to find the distance *r* − *r*^'^ at which the Ps-formation rate becomes a maximum.

It is assumed for simplicity that elastic collisions dominate all other processes near the source for energies *ε* ~ *ε*′. In this case, 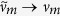 and the asymptotic solution of Eqn. (15) at high *v*′, together with Eqn. (25), yields


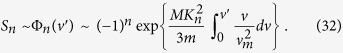


Hence, neglecting the contribution from the comparatively slowly varying constants *ρ*_*n*_, the solution of Eqn. (31) is


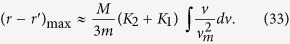


The values of *K*_1_ and *K*_2_ are found by solving the eligenvalue problem (12), and the integral in the right hand side evaluated approximately using the high energy momentum transfer cross section data from Refs 21, 22, 23, 24, 25. Thus, for liquid water of density *N* ≈ 3 × 10^28^ m^−3^ we find the range (in metres) is given by:


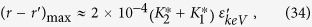


where 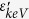 is the energy of the positrons at the source in units of keV, and the dimensionless eigenvalues 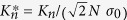, with *σ*_0_ = 10^−20^ m^2^.

## Results

**Figure 2:**
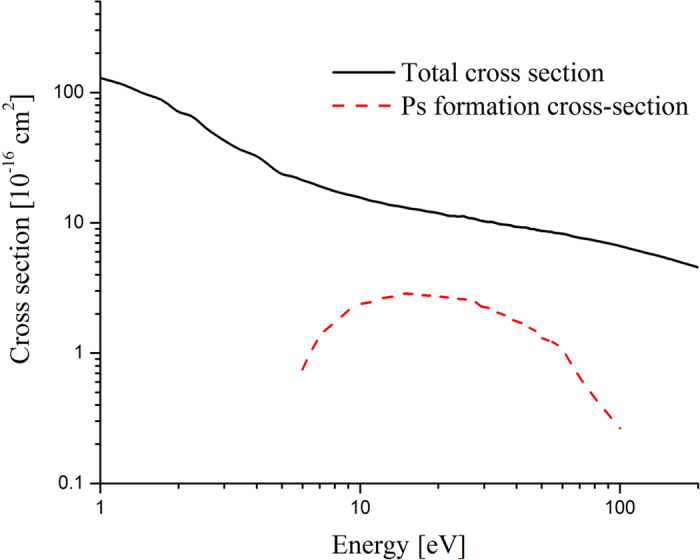
The total and Ps-formation cross-sections for positrons in water vapour. The full details of the cross-section used are detailed in^21^,^22^,^23^,^24^,^25^.

To this point, the formalism is quite general, the only restriction being non-relativistic kinetic theory is employed. In order to proceed with the calculation of the range numerical solution of the eigenvalue problem (12) is required. Cross-sections for the various positron scattering processes in the medium are required, in order to evaluate the collision terms in the collision operator (4) appearing in (12). In the case of a liquid water medium, sufficient cross-sections for a meaningful calculation are known only accurately below 100 eV, and this is the regime of focus for what follows. The best available sets have been detailed in^21^,^22^,^23^,^24^,^25^ and references therein, and the important elastic and Ps-formation cross-sections used in the current study are presented in Fig. 2. Note, no attempt is made to compare our results to relevant parameters from PET measurements in the current study. A comprehensive, more accurate analysis of positron transport over the entire energy range relevant to PET (i.e. from keV to thermal energies) would require all the open channel scattering cross-sections from thermal energies to 500 keV to be known, and such cross-section data is currently not available. When a kinematically complete set of cross-sections does become available, simulations of PET like conditions and associated comparisons will be addressed.

**Figure 3:**
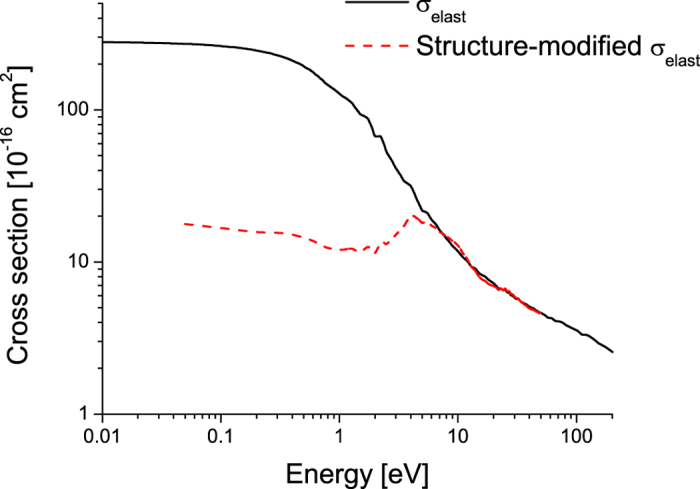
Impact of coherent scattering effects on the elastic momentum transfer cross-section for positron-water scattering. The static structure factor for water used in presented in Fig. 4^26^.

**Figure 4:**
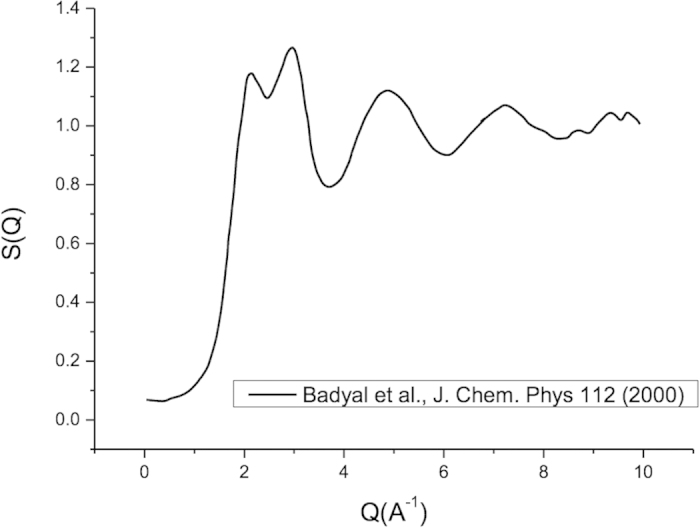
The static structure factor for liquid water used in the current study from Ref. 26.

In Fig. 3, we highlight the modification to the scattering cross-section induced by coherent scattering effects, where we have used the static structure of Badyal *et al.*^26^ (see Fig. 4) for the calculation. The effect of coherent scattering is to significantly reduce the momentum transfer cross-section in the low energy range less than 20 eV. Above that energy, the de Broglie wavelength is sufficiently small that coherent scattering effects are significantly reduced, and the cross-section approaches that for binary scattering. Note, there can be modifications to the scattering potential, but that is not considered in this study.

We again emphasize that the cross-section set is limited to energies well below those encountered near the source in PET. Therefore the numerical example discussed below might be seen as serving mainly to illustrate the general procedure which is to be followed using a more complete set of cross sections. In that spirit, we perform the calculations for a mono-energetic source of positrons emitted with speed *v*′ and energy *ε*′ = *mv*′^2^/2. The numerical calculation of eigenvalues is benchmarked against the analytic values of Parker [16] for the constant collision frequency model detailed in (18).

The lowest eigenvalues *K*_1_ and *K*_2_ for a liquid water medium (often considered as a surrogate for human tissue), as characterized by the cross sections in Refs 21, 22, 23, 24, 25, and the structure factor of Ref. 26, are shown in Table 1. For example, a mono-energetic positron source with *ε*′ ~ 3 keV, Eqn. (34) predicts a range of the order of approximately 1.2 mm. If the structure of the matter medium is ignored, then the eigenvalues are smaller, as shown in Table 1, and the estimate of the range as provided by Eqn. (34) is reduced by more than a factor of 2. This highlights the danger inherent in approximating a soft-condensed matter medium by a gas. Expanding the set to include higher energy processes will modify somewhat the eigenvalues of Table 1, and hence the estimate of the range, but the basic kinetic theoretical formalism outlined in the first part of this study remains the same.

We should emphasize here, that expression (34) is determined solely by the eigenvalues arising from solution of the eigenvalue problem (12). These eigenvalues are universal quantities and hence are independent of the boundary conditions.

## Concluding remarks

We have given an approximate estimate of the range of slow positrons in an idealized PET environment by solving Boltzmann’s equation incorporating the best available set of cross sections for low-energy positron-water scattering processes. For the first time, the explicit impact of accounting for the material structure and associated coherent scattering effects have been quantified and compared with the traditional ‘gas-phase’ assumption used in other contemporary work. The latter underestimates the positron range by more than a factor of 2 for the model used. The formalism given here can be readily extended to higher energies consistent with actual PET conditions, once the appropriate energy range of the corresponding cross sections becomes available. An important next step in this model is the treatment of Ps-formation (e.g. spur/blob models, possible clustering/rearrangement of water due to the presence of the positron) and associated Ps transport in a soft-condensed environment which would inform the issue of non-colinearity which also limits PET resolution.

## Additional Information

**How to cite this article**: Robson, R. E. *et al.* Positron kinetics in an idealized PET environment. *Sci. Rep.* **5**, 12674; doi: 10.1038/srep12674 (2015).

## Figures and Tables

**Table 1:** Dimensionless low order eigenvalues 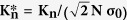 , with *σ*_0_ = 10^−20^ m^2^ for positrons in water using the cross-section set of Refs 21, 22, 23, 24, 25, and the static structure factor of Ref. 26.

Order	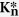 (gas phase)	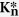 (liquid phase)
0	1.32 × 10^−8^	1.88 × 10^−7^
1	0.68	2.68
2	1.33	2.91

Since *N* ≈ 3 × 10^28^ m^3^ for liquid water, 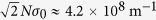.
